# Bis(4-cyano­phenolato)[hydro­tris­(3,5-dimethyl­pyrazol­yl)borato]nitro­syl­molybdenum(II)–4-hydroxy­benzonitrile–dichloro­methane (1/1/1)

**DOI:** 10.1107/S160053681004537X

**Published:** 2010-11-10

**Authors:** Mohammad B. Kassim, Jon A. McCleverty

**Affiliations:** a School of Chemistry, University of Bristol, Cantock Close, BS8 ITS Bristol, England

## Abstract

In the title compound, [Mo(C_15_H_22_BN_6_)(C_7_H_4_NO)_2_(NO)]·C_7_H_5_NO·CH_2_Cl_2_, the central Mo^II^ atom adopts a distorted *cis*-MoO_2_N_4_ octa­hedral geometry with the hydro­tris­(3,5-dimethyl­pyrazolylborate) anion attached to the metal in an *N*,*N*′,*N*′′-tridentate tripodal coordination mode. Two O-bonded 4-cyano­phenolate anions and a nitrosyl cation complete the coodination of the Mo^II^ atom. Two intra­molecular C—H⋯O and one C—H⋯N hydrogen bonds help to establish the configuration of the complex mol­ecule. The crystal structure is stabilized by inter­molecular C—H⋯N and C—H⋯O hydrogen bonds.

## Related literature

For related compounds, see: Kassim *et al.* (2002 ▶); Jones *et al.* (1997 ▶); Amoroso *et al.* (1994 ▶). For background to poly(pyrazol­yl)borate ligands, see: Trofimenko (1993 ▶).
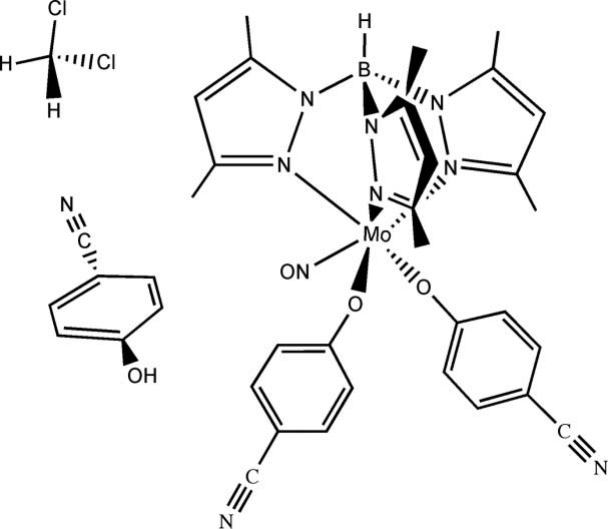


## Experimental

### 

#### Crystal data

[Mo(C_15_H_22_BN_6_)(C_7_H_4_NO)_2_(NO)]·C_7_H_5_NO·CH_2_Cl_2_
*M* *_r_* = 863.42
Triclinic, 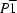

*a* = 11.9792 (19) Å
*b* = 12.630 (2) Å
*c* = 12.891 (2) Å
α = 90.120 (3)°
β = 92.459 (3)°
γ = 94.300 (3)°
*V* = 1943.0 (5) Å^3^
*Z* = 2
Mo *K*α radiation
μ = 0.53 mm^−1^
*T* = 173 K
0.18 × 0.10 × 0.05 mm

#### Data collection

Bruker SMART APEX CCD diffractometer
Absorption correction: multi-scan (*SADABS*; Bruker, 2000 ▶) *T*_min_ = 0.938, *T*_max_ = 0.974
19938 measured reflections
8844 independent reflections
5548 reflections with *I* > 2σ(*I*)
*R*_int_ = 0.066

#### Refinement

*R*[*F* ^2^ > 2σ(*F* ^2^)] = 0.052
*wR*(*F* ^2^) = 0.129
*S* = 0.98
8844 reflections
500 parameters
H atoms treated by a mixture of independent and constrained refinement
Δρ_max_ = 0.59 e Å^−3^
Δρ_min_ = −0.69 e Å^−3^

### 

Data collection: *SMART* (Bruker, 2000 ▶); cell refinement: *SAINT* (Bruker, 2000 ▶); data reduction: *SAINT*; program(s) used to solve structure: *SHELXS97* (Sheldrick, 2008 ▶); program(s) used to refine structure: *SHELXL97* (Sheldrick, 2008 ▶); molecular graphics: *PLATON* (Spek, 2009 ▶) and *SHELXTL* (Sheldrick, 2008 ▶); software used to prepare material for publication: *PLATON*.

## Supplementary Material

Crystal structure: contains datablocks global, I. DOI: 10.1107/S160053681004537X/hb5724sup1.cif

Structure factors: contains datablocks I. DOI: 10.1107/S160053681004537X/hb5724Isup2.hkl

Additional supplementary materials:  crystallographic information; 3D view; checkCIF report

## Figures and Tables

**Table 1:** Selected bond lengths (Å)

Mo1—N7	1.762 (4)
Mo1—O1	1.949 (3)
Mo1—O2	1.954 (3)
Mo1—N6	2.179 (3)
Mo1—N4	2.186 (3)
Mo1—N2	2.220 (3)

**Table 2:** Hydrogen-bond geometry (Å, °)

*D*—H⋯*A*	*D*—H	H⋯*A*	*D*⋯*A*	*D*—H⋯*A*
C5—H5*A*⋯O2	0.96	2.46	3.189 (5)	133
C10—H10*A*⋯N7	0.96	2.47	3.228 (6)	136
C15—H15*A*⋯N7	0.96	2.47	3.288 (6)	143
C4—H4*D*⋯O4^i^	0.96	2.52	3.360 (6)	146
C9—H9*B*⋯O3^ii^	0.96	2.37	3.219 (6)	147
C37—H37*B*⋯N9^iii^	0.96	2.53	3.366 (7)	144

Symmetry codes: (i) 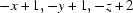; (ii) 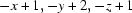; (iii) 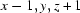.

**Fig. 1.**
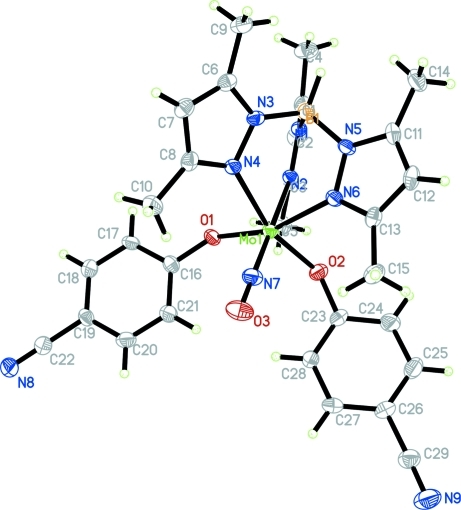
The complex in (I) with displacement ellipsoids drawn at the 50% probability level. H atoms are shown as spheres of arbitary radius.

**Fig. 2.**
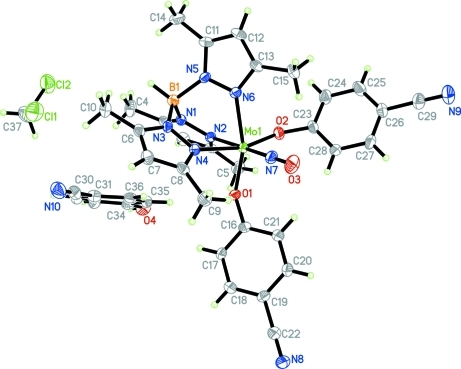
Molecules in the asymmetric unit with displacement ellipsoids drawn at the 50% probability level. H atoms are shown as spheres of arbitary radius.

**Fig. 3.**
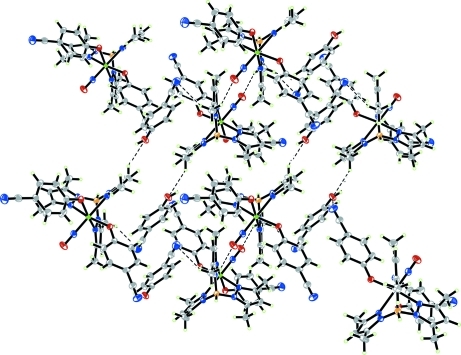
The packing diagram of the title compound showing the intermolecular H– bonds with dotted line with displacement ellipsoids drawn at the 50% probability level.

## supplementary crystallographic information

### Comment

Poly(pyrazolyl)borate ligands [Trofimenko (1993)] have attracted many researchers for the coordination chemistry of molybdenum complexes [Kassim *et al.* (2002), Jones *et al.* (1997) & Amoroso *et al.* (1994)]. In the title compound, (I), the hydrotris(3,5-dimethyl(pyrazolyl)borate ligand bonds to the central molybdenum atom in a tridentate manner through the N-atom at the 6-position of the pyrazolyl rings. Two 4-hydroxybenzonitrileate and a nitrosyl cation, bond *via* the O– and N-atom respectively, complete the octahedral coordination of the Mo(II) centre (Fig1). In addition, one molecule of the excess 4-hydroxybenzonitrile ligand and one molecule of CH_2_Cl_2_ solvent cystallized in the structure (Fig. 2).

The crystal structure is stabilized by three intramolecular hydrogen bonds C(5)—H(5 A)···O(2), C(10)—H(10 A)···N(7) and C(15)—H(15 A)···N(7). The crystal packing is stabilized by two C—H···O and one C—H···N intermolecular hydrogen bonds (Fig. 3).

### Experimental

The title compound was synthesized from a reaction of Mo(NO)Tp*Cl_2_ (0.1 mmol) with *p*-cyanophenol (0.25 mmol) in dichloromethane in the presence of triethylammine at refluxing temperature under N_2_ atmosphere (Kassim *et al.* 2002). Dark brown plates of (I) were obtained from a slow evaporation of dichloromethane solution of the title compound at room temperature. Yield 80%.

### Refinement

The H atoms attached to the B atom was located in a difference map, but those attached to carbon atoms were repositioned geometrically. The H atoms were initially refined with soft restraints on the bond lengths and angles to regularize their geometry (C—H in the range of 0.93–0.98, and O—H = 0.82 Å) and *U*_iso_(H) (in the range 1.2–1.5 times *U*_eq_ of the parent atom), after which the positions were refined with riding constraints.

### Crystal data

**Table:**

[Mo(C_15_H_22_BN_6_)(C_7_H_4_NO)_2_(NO)]·C_7_H_5_NO·CH_2_Cl_2_	*Z* = 2
*M**_r_* = 863.42	*F*(000) = 884
Triclinic, *P*1	*D*_x_ = 1.476 Mg m^−^^3^
Hall symbol: -P 1	Mo *K*α radiation, λ = 0.71073 Å
*a* = 11.9792 (19) Å	Cell parameters from 3353 reflections
*b* = 12.630 (2) Å	θ = 1.6–27.5°
*c* = 12.891 (2) Å	µ = 0.53 mm^−^^1^
α = 90.120 (3)°	*T* = 173 K
β = 92.459 (3)°	Plate, dark brown
γ = 94.300 (3)°	0.18 × 0.10 × 0.05 mm
*V* = 1943.0 (5) Å^3^	

### Data collection

**Table:**

Bruker SMART APEX CCD diffractometer	8844 independent reflections
Radiation source: fine-focus sealed tube	5548 reflections with *I* > 2σ(*I*)
Parallel, Graphite	*R*_int_ = 0.066
ω/2θ scans	θ_max_ = 27.5°, θ_min_ = 1.6°
Absorption correction: multi-scan (*SADABS*; Bruker, 2000)	*h* = −15→15
*T*_min_ = 0.938, *T*_max_ = 0.974	*k* = −16→16
19938 measured reflections	*l* = −16→16

### Refinement

**Table:**

Refinement on *F*^2^	Primary atom site location: structure-invariant direct methods
Least-squares matrix: full	Secondary atom site location: difference Fourier map
*R*[*F*^2^ > 2σ(*F*^2^)] = 0.052	Hydrogen site location: inferred from neighbouring sites
*wR*(*F*^2^) = 0.129	H atoms treated by a mixture of independent and constrained refinement
*S* = 0.98	*w* = 1/[σ^2^(*F*_o_^2^) + (0.0563*P*)^2^] where *P* = (*F*_o_^2^ + 2*F*_c_^2^)/3
8844 reflections	(Δ/σ)_max_ = 0.001
500 parameters	Δρ_max_ = 0.59 e Å^−^^3^
0 restraints	Δρ_min_ = −0.69 e Å^−^^3^

### Special details

**Table:**

Experimental. The crystal was placed in the cold stream of an Oxford Cryosystems open-flow nitrogen cryostat [Cosier, J. & Glazer, A. M. (1986). *J. Appl. Cryst.*19, 105–107] with a nominal stability of 0.1 K.
Geometry. All e.s.d.'s (except the e.s.d. in the dihedral angle between two l.s. planes) are estimated using the full covariance matrix. The cell e.s.d.'s are taken into account individually in the estimation of e.s.d.'s in distances, angles and torsion angles; correlations between e.s.d.'s in cell parameters are only used when they are defined by crystal symmetry. An approximate (isotropic) treatment of cell e.s.d.'s is used for estimating e.s.d.'s involving l.s. planes.
Refinement. Refinement of *F*^2^ against ALL reflections. The weighted *R*-factor *wR* and goodness of fit *S* are based on *F*^2^, conventional *R*-factors *R* are based on *F*, with *F* set to zero for negative *F*^2^. The threshold expression of *F*^2^ > σ(*F*^2^) is used only for calculating *R*-factors(gt) *etc*. and is not relevant to the choice of reflections for refinement. *R*-factors based on *F*^2^ are statistically about twice as large as those based on *F*, and *R*- factors based on ALL data will be even larger.

### Fractional atomic coordinates and isotropic or equivalent isotropic displacement parameters (Å^2^)

**Table:**

	*x*	*y*	*z*	*U*_iso_*/*U*_eq_	
Mo1	0.73371 (3)	0.79886 (3)	0.61020 (3)	0.01838 (11)	
Cl1	0.10962 (12)	0.73930 (12)	0.85992 (11)	0.0521 (4)	
Cl2	0.11742 (12)	0.51320 (11)	0.90460 (11)	0.0526 (4)	
O1	0.7960 (2)	0.8548 (2)	0.7431 (2)	0.0219 (6)	
O2	0.8338 (2)	0.6892 (2)	0.5782 (2)	0.0244 (7)	
O3	0.8373 (3)	0.9671 (3)	0.4762 (2)	0.0372 (8)	
O4	0.6205 (3)	0.6806 (2)	1.0784 (2)	0.0334 (8)	
H4A	0.5905	0.6522	1.1283	0.050*	
N1	0.5443 (3)	0.6553 (3)	0.7131 (2)	0.0190 (7)	
N2	0.6586 (3)	0.6735 (3)	0.7118 (2)	0.0187 (8)	
N3	0.4812 (3)	0.8253 (3)	0.6395 (2)	0.0192 (7)	
N4	0.5838 (3)	0.8832 (3)	0.6333 (2)	0.0195 (8)	
N5	0.5173 (3)	0.6799 (3)	0.5197 (2)	0.0203 (8)	
N6	0.6173 (3)	0.7294 (3)	0.4898 (2)	0.0204 (8)	
N7	0.7943 (3)	0.8988 (3)	0.5306 (3)	0.0250 (8)	
N8	1.1473 (3)	1.2538 (3)	0.9349 (3)	0.0345 (10)	
N9	1.2037 (4)	0.6758 (4)	0.2015 (3)	0.0447 (11)	
N10	0.2755 (3)	1.0246 (3)	0.8680 (3)	0.0385 (10)	
C1	0.5158 (4)	0.5900 (3)	0.7920 (3)	0.0224 (9)	
C2	0.6143 (4)	0.5627 (3)	0.8408 (3)	0.0265 (10)	
H2A	0.6209	0.5175	0.8972	0.032*	
C3	0.7015 (4)	0.6152 (3)	0.7898 (3)	0.0229 (9)	
C4	0.3968 (4)	0.5638 (4)	0.8183 (4)	0.0340 (11)	
H4B	0.3481	0.5955	0.7682	0.051*	
H4C	0.3848	0.5910	0.8862	0.051*	
H4D	0.3810	0.4882	0.8173	0.051*	
C5	0.8241 (4)	0.6155 (4)	0.8143 (3)	0.0307 (11)	
H5A	0.8640	0.6592	0.7652	0.046*	
H5B	0.8466	0.5443	0.8104	0.046*	
H5C	0.8407	0.6432	0.8831	0.046*	
C6	0.3999 (3)	0.8931 (3)	0.6482 (3)	0.0213 (9)	
C7	0.4492 (4)	0.9948 (3)	0.6481 (3)	0.0246 (10)	
H7A	0.4133	1.0572	0.6537	0.029*	
C8	0.5630 (4)	0.9862 (3)	0.6380 (3)	0.0225 (9)	
C9	0.2803 (3)	0.8542 (4)	0.6581 (3)	0.0294 (10)	
H9A	0.2734	0.7780	0.6553	0.044*	
H9B	0.2357	0.8818	0.6022	0.044*	
H9C	0.2546	0.8777	0.7232	0.044*	
C10	0.6525 (4)	1.0744 (4)	0.6366 (3)	0.0318 (11)	
H10A	0.7238	1.0457	0.6293	0.048*	
H10B	0.6537	1.1140	0.7003	0.048*	
H10C	0.6379	1.1204	0.5792	0.048*	
C11	0.4625 (4)	0.6304 (3)	0.4370 (3)	0.0224 (9)	
C12	0.5288 (4)	0.6482 (3)	0.3529 (3)	0.0253 (10)	
H12A	0.5131	0.6230	0.2856	0.030*	
C13	0.6228 (4)	0.7104 (3)	0.3872 (3)	0.0214 (9)	
C14	0.3518 (4)	0.5694 (4)	0.4444 (3)	0.0322 (11)	
H14A	0.3280	0.5725	0.5145	0.048*	
H14B	0.3585	0.4967	0.4252	0.048*	
H14C	0.2978	0.5996	0.3985	0.048*	
C15	0.7181 (4)	0.7532 (4)	0.3242 (3)	0.0288 (11)	
H15A	0.7720	0.7940	0.3682	0.043*	
H15B	0.6903	0.7977	0.2701	0.043*	
H15C	0.7531	0.6953	0.2938	0.043*	
C16	0.8660 (3)	0.9372 (3)	0.7777 (3)	0.0209 (9)	
C17	0.8478 (3)	0.9804 (3)	0.8746 (3)	0.0253 (10)	
H17A	0.7876	0.9534	0.9123	0.030*	
C18	0.9183 (4)	1.0624 (4)	0.9145 (3)	0.0286 (10)	
H18A	0.9050	1.0915	0.9788	0.034*	
C19	1.0096 (3)	1.1025 (3)	0.8595 (3)	0.0227 (9)	
C20	1.0283 (4)	1.0589 (4)	0.7636 (3)	0.0291 (11)	
H20A	1.0892	1.0851	0.7265	0.035*	
C21	0.9575 (4)	0.9774 (4)	0.7231 (3)	0.0309 (11)	
H21A	0.9706	0.9487	0.6586	0.037*	
C22	1.0870 (4)	1.1871 (4)	0.9021 (3)	0.0283 (10)	
C23	0.9139 (3)	0.6850 (3)	0.5062 (3)	0.0228 (9)	
C24	0.9115 (4)	0.5955 (4)	0.4432 (3)	0.0314 (11)	
H24A	0.8589	0.5388	0.4531	0.038*	
C25	0.9872 (4)	0.5906 (4)	0.3657 (3)	0.0338 (11)	
H25A	0.9849	0.5310	0.3230	0.041*	
C26	1.0665 (4)	0.6743 (4)	0.3515 (3)	0.0261 (10)	
C27	1.0730 (4)	0.7615 (4)	0.4182 (4)	0.0342 (11)	
H27A	1.1277	0.8169	0.4105	0.041*	
C28	0.9978 (4)	0.7648 (4)	0.4957 (3)	0.0323 (11)	
H28A	1.0036	0.8218	0.5418	0.039*	
C29	1.1427 (4)	0.6732 (4)	0.2677 (4)	0.0317 (11)	
C30	0.3336 (4)	0.9645 (4)	0.9018 (3)	0.0302 (11)	
C31	0.4072 (4)	0.8889 (4)	0.9451 (3)	0.0268 (10)	
C32	0.3746 (4)	0.8264 (4)	1.0294 (3)	0.0317 (11)	
H32A	0.3044	0.8317	1.0562	0.038*	
C33	0.4462 (4)	0.7573 (4)	1.0728 (3)	0.0304 (11)	
H33A	0.4241	0.7159	1.1290	0.036*	
C34	0.5512 (4)	0.7487 (3)	1.0335 (3)	0.0257 (10)	
C35	0.5824 (4)	0.8075 (3)	0.9467 (3)	0.0243 (10)	
H35A	0.6512	0.7993	0.9182	0.029*	
C36	0.5121 (4)	0.8775 (3)	0.9033 (3)	0.0238 (10)	
H36A	0.5338	0.9174	0.8460	0.029*	
C37	0.1033 (5)	0.6406 (4)	0.9550 (4)	0.0489 (14)	
H37A	0.0322	0.6409	0.9882	0.059*	
H37B	0.1625	0.6570	1.0076	0.059*	
B1	0.4723 (4)	0.7041 (4)	0.6283 (4)	0.0223 (11)	
H1	0.386 (3)	0.671 (3)	0.629 (3)	0.009 (9)*	

### Atomic displacement parameters (Å^2^)

**Table:**

	*U*^11^	*U*^22^	*U*^33^	*U*^12^	*U*^13^	*U*^23^
Mo1	0.01678 (19)	0.0215 (2)	0.01643 (18)	−0.00219 (14)	0.00264 (13)	−0.00082 (13)
Cl1	0.0532 (9)	0.0483 (9)	0.0535 (8)	−0.0093 (7)	0.0112 (7)	0.0095 (7)
Cl2	0.0503 (9)	0.0440 (9)	0.0645 (9)	0.0010 (7)	0.0197 (7)	−0.0023 (7)
O1	0.0210 (16)	0.0221 (16)	0.0215 (15)	−0.0056 (13)	0.0017 (12)	−0.0017 (12)
O2	0.0220 (16)	0.0300 (18)	0.0217 (15)	0.0035 (14)	0.0046 (12)	−0.0013 (13)
O3	0.035 (2)	0.038 (2)	0.0381 (19)	−0.0068 (16)	0.0096 (15)	0.0151 (16)
O4	0.0366 (19)	0.0328 (19)	0.0303 (17)	0.0024 (16)	−0.0029 (14)	0.0124 (14)
N1	0.0199 (19)	0.0192 (19)	0.0177 (17)	−0.0026 (15)	0.0047 (14)	0.0002 (14)
N2	0.0184 (18)	0.022 (2)	0.0157 (17)	−0.0024 (15)	0.0015 (14)	0.0001 (14)
N3	0.0163 (18)	0.024 (2)	0.0171 (17)	0.0007 (15)	0.0036 (13)	0.0012 (14)
N4	0.0188 (18)	0.022 (2)	0.0171 (17)	−0.0017 (15)	0.0039 (14)	−0.0011 (14)
N5	0.0166 (18)	0.025 (2)	0.0187 (17)	−0.0049 (15)	0.0003 (14)	0.0000 (14)
N6	0.0188 (19)	0.022 (2)	0.0196 (17)	−0.0021 (15)	0.0004 (14)	−0.0010 (14)
N7	0.021 (2)	0.031 (2)	0.0217 (19)	−0.0033 (17)	0.0019 (15)	0.0013 (16)
N8	0.025 (2)	0.032 (2)	0.045 (2)	−0.0007 (19)	0.0017 (18)	−0.0116 (19)
N9	0.041 (3)	0.053 (3)	0.041 (3)	0.003 (2)	0.018 (2)	0.003 (2)
N10	0.039 (3)	0.040 (3)	0.039 (2)	0.014 (2)	0.0002 (19)	−0.005 (2)
C1	0.029 (2)	0.021 (2)	0.018 (2)	−0.0006 (19)	0.0079 (17)	−0.0010 (17)
C2	0.034 (3)	0.022 (2)	0.024 (2)	0.004 (2)	0.0029 (19)	0.0078 (18)
C3	0.033 (3)	0.017 (2)	0.019 (2)	0.0032 (19)	−0.0033 (18)	−0.0047 (17)
C4	0.029 (3)	0.029 (3)	0.045 (3)	0.003 (2)	0.015 (2)	0.013 (2)
C5	0.026 (3)	0.034 (3)	0.032 (3)	0.005 (2)	−0.0042 (19)	0.004 (2)
C6	0.022 (2)	0.027 (2)	0.015 (2)	0.0038 (19)	0.0023 (16)	0.0015 (17)
C7	0.027 (2)	0.023 (2)	0.024 (2)	0.007 (2)	0.0040 (18)	0.0026 (18)
C8	0.030 (2)	0.023 (2)	0.015 (2)	0.002 (2)	0.0050 (17)	0.0008 (17)
C9	0.022 (2)	0.032 (3)	0.035 (3)	0.004 (2)	0.0073 (19)	0.003 (2)
C10	0.034 (3)	0.026 (3)	0.035 (3)	−0.006 (2)	0.009 (2)	0.000 (2)
C11	0.028 (2)	0.017 (2)	0.022 (2)	0.0038 (19)	−0.0070 (18)	−0.0025 (17)
C12	0.038 (3)	0.024 (2)	0.015 (2)	0.007 (2)	−0.0043 (18)	−0.0023 (17)
C13	0.030 (2)	0.020 (2)	0.015 (2)	0.0048 (19)	0.0010 (17)	0.0008 (16)
C14	0.029 (3)	0.032 (3)	0.034 (3)	−0.003 (2)	−0.007 (2)	−0.006 (2)
C15	0.032 (3)	0.035 (3)	0.019 (2)	0.003 (2)	0.0080 (18)	−0.0003 (19)
C16	0.021 (2)	0.021 (2)	0.020 (2)	−0.0004 (18)	−0.0035 (17)	0.0007 (17)
C17	0.020 (2)	0.031 (3)	0.024 (2)	−0.007 (2)	0.0060 (17)	−0.0032 (19)
C18	0.030 (3)	0.029 (3)	0.026 (2)	−0.001 (2)	0.0045 (19)	−0.0070 (19)
C19	0.019 (2)	0.017 (2)	0.032 (2)	0.0022 (18)	−0.0036 (18)	−0.0014 (18)
C20	0.024 (2)	0.037 (3)	0.026 (2)	−0.003 (2)	0.0072 (18)	−0.003 (2)
C21	0.029 (3)	0.036 (3)	0.026 (2)	−0.009 (2)	0.0074 (19)	−0.010 (2)
C22	0.027 (3)	0.028 (3)	0.031 (2)	0.002 (2)	0.0023 (19)	−0.004 (2)
C23	0.021 (2)	0.028 (3)	0.020 (2)	0.0064 (19)	0.0034 (17)	−0.0008 (18)
C24	0.032 (3)	0.024 (3)	0.038 (3)	−0.003 (2)	0.011 (2)	0.001 (2)
C25	0.041 (3)	0.030 (3)	0.032 (3)	0.005 (2)	0.011 (2)	−0.005 (2)
C26	0.024 (2)	0.032 (3)	0.024 (2)	0.007 (2)	0.0059 (18)	0.0026 (19)
C27	0.021 (2)	0.037 (3)	0.044 (3)	−0.005 (2)	0.011 (2)	−0.007 (2)
C28	0.020 (2)	0.039 (3)	0.037 (3)	−0.003 (2)	0.0048 (19)	−0.018 (2)
C29	0.032 (3)	0.027 (3)	0.037 (3)	0.004 (2)	0.010 (2)	0.004 (2)
C30	0.030 (3)	0.037 (3)	0.024 (2)	0.003 (2)	0.005 (2)	−0.004 (2)
C31	0.031 (3)	0.024 (3)	0.025 (2)	0.001 (2)	−0.0023 (19)	−0.0020 (19)
C32	0.027 (3)	0.040 (3)	0.028 (2)	−0.003 (2)	0.0056 (19)	0.000 (2)
C33	0.030 (3)	0.035 (3)	0.026 (2)	−0.001 (2)	0.0057 (19)	0.010 (2)
C34	0.028 (2)	0.026 (3)	0.023 (2)	−0.001 (2)	−0.0053 (18)	0.0008 (18)
C35	0.024 (2)	0.025 (2)	0.024 (2)	0.000 (2)	0.0036 (18)	−0.0016 (18)
C36	0.028 (2)	0.024 (2)	0.018 (2)	−0.002 (2)	−0.0011 (18)	−0.0032 (17)
C37	0.067 (4)	0.043 (3)	0.036 (3)	−0.004 (3)	0.010 (3)	0.000 (2)
B1	0.022 (3)	0.022 (3)	0.022 (2)	0.000 (2)	0.003 (2)	−0.002 (2)

### Geometric parameters (Å, °)

**Table:**

Mo1—N7	1.762 (4)	C10—H10C	0.9600
Mo1—O1	1.949 (3)	C11—C12	1.380 (6)
Mo1—O2	1.954 (3)	C11—C14	1.489 (6)
Mo1—N6	2.179 (3)	C12—C13	1.380 (6)
Mo1—N4	2.186 (3)	C12—H12A	0.9300
Mo1—N2	2.220 (3)	C13—C15	1.498 (6)
Cl1—C37	1.750 (5)	C14—H14A	0.9600
Cl2—C37	1.757 (5)	C14—H14B	0.9600
O1—C16	1.348 (5)	C14—H14C	0.9600
O2—C23	1.366 (5)	C15—H15A	0.9600
O3—N7	1.211 (4)	C15—H15B	0.9600
O4—C34	1.353 (5)	C15—H15C	0.9600
O4—H4A	0.8200	C16—C21	1.392 (6)
N1—C1	1.350 (5)	C16—C17	1.395 (5)
N1—N2	1.372 (4)	C17—C18	1.372 (6)
N1—B1	1.523 (6)	C17—H17A	0.9300
N2—C3	1.354 (5)	C18—C19	1.394 (6)
N3—C6	1.352 (5)	C18—H18A	0.9300
N3—N4	1.387 (4)	C19—C20	1.386 (6)
N3—B1	1.532 (6)	C19—C22	1.452 (6)
N4—C8	1.345 (5)	C20—C21	1.372 (6)
N5—C11	1.355 (5)	C20—H20A	0.9300
N5—N6	1.379 (4)	C21—H21A	0.9300
N5—B1	1.560 (5)	C23—C28	1.380 (6)
N6—C13	1.349 (5)	C23—C24	1.389 (6)
N8—C22	1.136 (5)	C24—C25	1.382 (6)
N9—C29	1.146 (5)	C24—H24A	0.9300
N10—C30	1.143 (6)	C25—C26	1.385 (6)
C1—C2	1.379 (6)	C25—H25A	0.9300
C1—C4	1.493 (6)	C26—C27	1.391 (6)
C2—C3	1.386 (6)	C26—C29	1.445 (6)
C2—H2A	0.9300	C27—C28	1.377 (6)
C3—C5	1.489 (6)	C27—H27A	0.9300
C4—H4B	0.9600	C28—H28A	0.9300
C4—H4C	0.9600	C30—C31	1.444 (7)
C4—H4D	0.9600	C31—C32	1.397 (6)
C5—H5A	0.9600	C31—C36	1.405 (6)
C5—H5B	0.9600	C32—C33	1.372 (6)
C5—H5C	0.9600	C32—H32A	0.9300
C6—C7	1.373 (6)	C33—C34	1.388 (6)
C6—C9	1.491 (6)	C33—H33A	0.9300
C7—C8	1.388 (6)	C34—C35	1.393 (6)
C7—H7A	0.9300	C35—C36	1.370 (6)
C8—C10	1.488 (6)	C35—H35A	0.9300
C9—H9A	0.9600	C36—H36A	0.9300
C9—H9B	0.9600	C37—H37A	0.9700
C9—H9C	0.9600	C37—H37B	0.9700
C10—H10A	0.9600	B1—H1	1.09 (4)
C10—H10B	0.9600		
			
N7—Mo1—O1	97.65 (13)	N6—C13—C12	109.4 (4)
N7—Mo1—O2	97.14 (14)	N6—C13—C15	123.2 (4)
O1—Mo1—O2	102.85 (11)	C12—C13—C15	127.5 (4)
N7—Mo1—N6	95.33 (14)	C11—C14—H14A	109.5
O1—Mo1—N6	162.05 (11)	C11—C14—H14B	109.5
O2—Mo1—N6	87.73 (12)	H14A—C14—H14B	109.5
N7—Mo1—N4	93.57 (14)	C11—C14—H14C	109.5
O1—Mo1—N4	88.99 (12)	H14A—C14—H14C	109.5
O2—Mo1—N4	162.77 (12)	H14B—C14—H14C	109.5
N6—Mo1—N4	77.84 (12)	C13—C15—H15A	109.5
N7—Mo1—N2	179.42 (15)	C13—C15—H15B	109.5
O1—Mo1—N2	81.80 (11)	H15A—C15—H15B	109.5
O2—Mo1—N2	82.84 (12)	C13—C15—H15C	109.5
N6—Mo1—N2	85.25 (12)	H15A—C15—H15C	109.5
N4—Mo1—N2	86.58 (12)	H15B—C15—H15C	109.5
C16—O1—Mo1	137.6 (2)	O1—C16—C21	122.9 (4)
C23—O2—Mo1	131.6 (3)	O1—C16—C17	117.9 (4)
C34—O4—H4A	109.5	C21—C16—C17	119.1 (4)
C1—N1—N2	110.3 (3)	C18—C17—C16	120.2 (4)
C1—N1—B1	130.9 (4)	C18—C17—H17A	119.9
N2—N1—B1	118.8 (3)	C16—C17—H17A	119.9
C3—N2—N1	106.4 (3)	C17—C18—C19	120.5 (4)
C3—N2—Mo1	133.0 (3)	C17—C18—H18A	119.8
N1—N2—Mo1	119.9 (2)	C19—C18—H18A	119.8
C6—N3—N4	109.1 (3)	C20—C19—C18	119.3 (4)
C6—N3—B1	130.0 (3)	C20—C19—C22	119.6 (4)
N4—N3—B1	120.6 (3)	C18—C19—C22	121.1 (4)
C8—N4—N3	106.5 (3)	C21—C20—C19	120.4 (4)
C8—N4—Mo1	134.3 (3)	C21—C20—H20A	119.8
N3—N4—Mo1	119.1 (2)	C19—C20—H20A	119.8
C11—N5—N6	109.8 (3)	C20—C21—C16	120.5 (4)
C11—N5—B1	128.8 (4)	C20—C21—H21A	119.7
N6—N5—B1	120.1 (3)	C16—C21—H21A	119.7
C13—N6—N5	106.5 (3)	N8—C22—C19	179.4 (5)
C13—N6—Mo1	134.9 (3)	O2—C23—C28	122.6 (4)
N5—N6—Mo1	118.1 (2)	O2—C23—C24	118.2 (4)
O3—N7—Mo1	179.1 (3)	C28—C23—C24	119.2 (4)
N1—C1—C2	107.0 (4)	C25—C24—C23	120.0 (4)
N1—C1—C4	122.2 (4)	C25—C24—H24A	120.0
C2—C1—C4	130.6 (4)	C23—C24—H24A	120.0
C1—C2—C3	107.1 (4)	C24—C25—C26	120.1 (4)
C1—C2—H2A	126.5	C24—C25—H25A	119.9
C3—C2—H2A	126.5	C26—C25—H25A	119.9
N2—C3—C2	109.1 (4)	C25—C26—C27	119.9 (4)
N2—C3—C5	122.4 (4)	C25—C26—C29	121.1 (4)
C2—C3—C5	128.5 (4)	C27—C26—C29	119.0 (4)
C1—C4—H4B	109.5	C28—C27—C26	119.3 (4)
C1—C4—H4C	109.5	C28—C27—H27A	120.4
H4B—C4—H4C	109.5	C26—C27—H27A	120.4
C1—C4—H4D	109.5	C27—C28—C23	121.2 (4)
H4B—C4—H4D	109.5	C27—C28—H28A	119.4
H4C—C4—H4D	109.5	C23—C28—H28A	119.4
C3—C5—H5A	109.5	N9—C29—C26	177.8 (5)
C3—C5—H5B	109.5	N10—C30—C31	179.6 (5)
H5A—C5—H5B	109.5	C32—C31—C36	119.3 (4)
C3—C5—H5C	109.5	C32—C31—C30	120.1 (4)
H5A—C5—H5C	109.5	C36—C31—C30	120.6 (4)
H5B—C5—H5C	109.5	C33—C32—C31	120.0 (4)
N3—C6—C7	108.1 (4)	C33—C32—H32A	120.0
N3—C6—C9	121.6 (4)	C31—C32—H32A	120.0
C7—C6—C9	130.2 (4)	C32—C33—C34	120.5 (4)
C6—C7—C8	106.5 (4)	C32—C33—H33A	119.7
C6—C7—H7A	126.7	C34—C33—H33A	119.7
C8—C7—H7A	126.7	O4—C34—C33	119.3 (4)
N4—C8—C7	109.7 (4)	O4—C34—C35	121.0 (4)
N4—C8—C10	123.2 (4)	C33—C34—C35	119.7 (4)
C7—C8—C10	127.1 (4)	C36—C35—C34	120.3 (4)
C6—C9—H9A	109.5	C36—C35—H35A	119.9
C6—C9—H9B	109.5	C34—C35—H35A	119.9
H9A—C9—H9B	109.5	C35—C36—C31	120.1 (4)
C6—C9—H9C	109.5	C35—C36—H36A	120.0
H9A—C9—H9C	109.5	C31—C36—H36A	120.0
H9B—C9—H9C	109.5	Cl1—C37—Cl2	112.9 (3)
C8—C10—H10A	109.5	Cl1—C37—H37A	109.0
C8—C10—H10B	109.5	Cl2—C37—H37A	109.0
H10A—C10—H10B	109.5	Cl1—C37—H37B	109.0
C8—C10—H10C	109.5	Cl2—C37—H37B	109.0
H10A—C10—H10C	109.5	H37A—C37—H37B	107.8
H10B—C10—H10C	109.5	N1—B1—N3	110.1 (3)
N5—C11—C12	107.0 (4)	N1—B1—N5	109.8 (3)
N5—C11—C14	122.7 (4)	N3—B1—N5	106.2 (3)
C12—C11—C14	130.3 (4)	N1—B1—H1	111.3 (19)
C13—C12—C11	107.3 (4)	N3—B1—H1	111.6 (19)
C13—C12—H12A	126.4	N5—B1—H1	107.7 (18)
C11—C12—H12A	126.4		

### Hydrogen-bond geometry (Å, °)

**Table:**

*D*—H···*A*	*D*—H	H···*A*	*D*···*A*	*D*—H···*A*
C5—H5A···O2	0.96	2.46	3.189 (5)	133
C10—H10A···N7	0.96	2.47	3.228 (6)	136
C15—H15A···N7	0.96	2.47	3.288 (6)	143
C4—H4D···O4^i^	0.96	2.52	3.360 (6)	146
C9—H9B···O3^ii^	0.96	2.37	3.219 (6)	147
C37—H37B···N9^iii^	0.96	2.53	3.366 (7)	144

Symmetry codes:  (i) −*x*+1, −*y*+1, −*z*+2; (ii) −*x*+1, −*y*+2, −*z*+1; (iii) *x*−1, *y*, *z*+1.

## References

[bb2] Amoroso, A. J., Cargill Thompson, A. M., Jeffery, J. C., Jones, P. L., McCleverty, J. A. & Ward, M. D. (1994). *J. Chem. Soc. Chem. Commun.* pp. 2751–2752.

[bb4] Bruker (2000). *SADABS*, *SMART* and *SAINT* Bruker AXS Inc., Madison, Wisconsin, USA.

[bb6] Jones, P. L., Amoroso, A. J., Jeffery, J. C., McCleverty, J. A., Psillakis, E., Rees, L. H. & Ward, M. D. (1997). *Inorg. Chem.* **36**, 10–18.

[bb7] Kassim, M. B., Paul, R. L., Jeffery, J. C., McCleverty, J. A. & Ward, M. D. (2002). *Inorg. Chim. Acta*, **327**, 160–168.

[bb8] Sheldrick, G. M. (2008). *Acta Cryst.* A**64**, 112–122. 10.1107/S0108767307043930 18156677

[bb9] Spek, A. L. (2009). *Acta Cryst.* D**65**, 148–155. 10.1107/S090744490804362X PMC2631630 19171970

[bb10] Trofimenko, S. (1993). *Chem. Rev.* **93**, 943–980.

